# Heart Rate Variability and Inflammatory Stress Response in Young African American Men: Implications for Cardiovascular Risk

**DOI:** 10.3389/fcvm.2021.745864

**Published:** 2021-10-13

**Authors:** Dina Tell, Robert L. Burr, Herbert L. Mathews, Linda Witek Janusek

**Affiliations:** ^1^Department of Health Systems and Adult Health, Marcella Niehoff School of Nursing, Loyola University Chicago, Chicago, IL, United States; ^2^Department of Biobehavioral Nursing and Health Informatics, School of Nursing, University of Washington, Seattle, WA, United States; ^3^Department of Microbiology and Immunology, Stritch School of Medicine, Loyola University Chicago, Chicago, IL, United States; ^4^Department of Health Promotion, Marcella Niehoff School of Nursing, Loyola University Chicago, Chicago, IL, United States

**Keywords:** childhood adversity, inflammation, stress, heart rate variability, stress response

## Abstract

**Background:** African American men have a disproportionately higher incidence of and suffer greater severity and earlier death from cardiovascular disease (CVD). A common feature of many diseases, which disproportionately afflict disadvantaged African Americans, is inflammation. In particular, inflammation plays a decisive role in the pathogenesis of CVD in that persistent inflammation contributes to plaque evolution and destabilization. Adverse childhood experiences increase the risk for adult inflammatory based disease, particularly cardiovascular disease. This inflammatory burden becomes evident during stressful events and may be related to alterations in autonomic nervous system (ANS) activity. We previously reported that African American men who experienced childhood adversity exhibited a greater inflammatory (IL-6) response to acute stress challenge (Trier Social Stress Test – TSST). The purpose of this study was to determine whether altered ANS activity, as measured by heart rate variability (HRV), contributes to a greater proinflammatory response to stress in those exposed to childhood adversity.

**Methods:** Thirty-four African American adult males underwent the TSST while instrumented with Holter monitors to record continuous heart rate for HRV determination. HRV was calculated as the low frequency (LF) to high frequency (HF) heart rate ratio (LF/HF), with higher LF/HF ratios corresponding to higher sympathetic vs. parasympathetic activity. Salivary samples were collected pre- and post-TSST to measure the proinflammatory cytokine IL-6. Childhood adversity was assessed by the Childhood Trauma Questionnaire.

**Results:** Hierarchical linear modeling demonstrated that higher levels of physical abuse were related to a steeper rise in LF/HF ratio during the TSST. Further, a higher LF/HF ratio, in combination with greater exposure to emotional and physical abuse was associated with a greater IL-6 response to the TSST.

**Conclusions:** These findings suggest that adverse childhood experiences associate with an adult phenotype characterized by an altered ANS response to stress as well as a greater proinflammatory (IL-6) response to an acute stressor. Elevations in salivary inflammatory markers have been associated with increased CVD risk. In conclusion, these findings suggest a role for the ANS in the underlying neuro-biological processes whereby childhood adversity predisposes to a more intense inflammatory response to stressful challenge during adulthood.

## Introduction

African American men have a disproportionately higher incidence of and suffer greater severity and earlier death from cardiovascular disease (CVD) (1). The landmark Adverse Childhood Experience (ACE) study (2), and follow-up studies (3, 4), revealed that increasing exposure to ACEs increase risk for poor adult health, such as higher blood pressure, poorer metabolic profiles, greater inflammation, and premature death. A common feature of many diseases, which disproportionately afflict disadvantaged African Americans (5), is inflammation. In particular, inflammation plays a decisive role in the pathogenesis of CVD in that persistent inflammation contributes to plaque evolution and destabilization (6). Exposure to early life adversity predicts greater inflammation in African Americans than in whites (7), which may increase risk for inflammatory-based disease in disadvantaged African Americans (5). Adversity during early life, including childhood, is theorized to imprint developing stress response systems, resulting in an adult proinflammatory phenotype (3) characterized by chronic low grade inflammation (8, 9) and an exaggerated inflammatory response to acute stress challenge (10). Moreover, inflammatory risk subsequent to early life adversity emerges early in life, as it has been observed in adolescents and young adults (11). Although young adults do not typically exhibit traditional signs of disease, many harbor insidious processes that increase future disease risk. For example, growing numbers of American youth exhibit preclinical signs of CVD, which cluster by socioeconomic status (SES) (12). Forty percent of African American adolescents have clinically defined pre-hypertension (13), and greater incidence of central adiposity, high blood pressure, insulin resistance, and subclinical atherosclerosis (14–17). Thus, it is important to explore the underlying neuro-biological processes whereby the experience of adversity in childhood predisposes to a more intense inflammatory response to stressful challenge during adulthood.

Childhood adversity is associated with persistent changes in the balance of the autonomic nervous system (ANS), characterized by lower parasympathetic (i.e., vagal) activity (18). Modulation of vagal nerve activity may influence the proinflammatory response to stress, through reflex activation of the cholinergic anti-inflammatory pathway, which produces a rapid anti-inflammatory response (19–21). In response to inflammation, stimulation of the vagus nerve results in a subsequent cholinergic signal that inhibits proinflammatory cytokine synthesis (20, 22–24). Several lines of evidence in animal models demonstrate that activation of vagal pathways inhibits proinflammatory cytokine release, dampening inflammation through the cholinergic anti-inflammatory pathway (25–27).

Heart rate variability (HRV) is a non-invasive approach to measure ANS activity by power spectral analysis of the heart rate time series (28). HRV provides an indirect assessment of ANS balance, such that lower HRV (high-frequency domain; HF) indicates decreased parasympathetic modulation of the heart (29). Evidence in humans, using high frequency-HRV as an index of parasympathetic activity, is consistent with the concept that greater parasympathetic activity is related to lower stimulated production of the proinflammatory cytokines, IL-6 and TNF-alpha (30), and lower circulating C-reactive protein (CRP), and IL-6 levels (31–33). Recent meta-analysis of human studies evaluating HRV and inflammation supports a negative relationship between HRV indices (i.e., HF domain, Standard Deviation of NN intervals or SDNN Index) and inflammation consistent with the concept that these indices of HRV reflect activity of the neurophysiological pathway involved in adaptively regulating inflammatory processes in humans (34).

We previously reported that emerging adult African American men who had greater exposure to childhood adversity exhibited a greater salivary IL-6 response to acute stress (Trier Social Stress Test, TSST) (35). In this same cohort of African American men, we used HRV analysis to examine whether variations in the ANS response to stress challenge related to childhood adversity-related amplification of the IL-6 response to the TSST. Salivary IL-6 was measured in that inflammation within the mouth has been linked to increased risk for CVD (36), including increased risk for carotid atherosclerosis (37). We measured HRV and computed low frequency (LF) and high frequency (HF) ratio (LF/HF) to assess the ANS response to acute stress (28). We hypothesized that childhood adversity would be associated with an altered ANS response to stress; and, further these adversity factors would predict the IL-6 response to stress. In addition, we examined the interaction between childhood adversity and ANS reactivity in relation to the IL-6 response. Social support and health behaviors were evaluated as co-variates to understand their influence on the relationship between childhood adversity and stress reactivity.

## Methods

### Participants

We analyzed HRV data in the context of childhood adversity and the salivary IL-6 response to the TSST in African American males who participated in our previous study (35). The men (*N* = 34) were between the ages of 18 and 27 (mean age = 20.2 ± 2.3 years) and were recruited from two Chicago metropolitan low-income neighborhoods, with a portion of the sample recruited from community centers that provide resources to low-income African American families. Enrollment eligibility was established by a brief self-report questionnaire.

Screening was based on a brief interview with the research study nurse prior to the enrollment. Potential participants were asked to self-report their current health history and use of prescription medication, drugs and alcohol. Participants were excluded if they had reported any of the following: acute illness or/and were hospitalized within 2 weeks of the recruitment interview, current major psychiatric disorders, autoimmune disease, heart disease, current drug or alcohol abuse, and use of psychotropic or anti-inflammatory medications. Participants were also excluded if they did not comply with the following criteria: no smoking within 24 h, no alcohol within 12 h, caffeine, or rigorous exercise within 4 h on the day of the study.

A sample size of 40 participants was projected to complete the study. A prospective power analysis was conducted to estimate sample size needed to achieve 80% statistical power to detect an effect size of *f* ^2^ = 0.40 using multivariate regression with a 0.05 two-sided level of significance. However, due to the recruitment challenges, the total sample of participants was *N* = 34.

### Procedure

This study was approved by the Loyola University Chicago Institutional Review Board for the Protection of Human Subjects and informed consent was obtained from all participants. The work was carried out in accordance with The Code of Ethics of the World Medical Association, Declaration of Helsinki. Consented participants were scheduled between 8:00 a.m. and 10:00 a.m. to undergo the TSST, which is an established protocol used to induce psychological stress (38, 39). Prior to the TSST protocol, participants were asked to avoid caffeine for 4 h, alcohol and exercise for 12 h and smoking and over-the-counter medications for 24 h. Height and weight were measured to determine body mass index (BMI). Participants were instrumented with a Holter monitor to record heart rate throughout the procedure for determination of HRV. After the Holter monitor was attached and activated participants sat quietly for 15 min to obtain a baseline (T0) saliva sample for IL-6 determination. The TSST was then administered by two evaluative judges. For the TSST, the participants delivered a 4-min impromptu speech to simulate a job interview, followed by a 4-min mental arithmetic task. At completion of the TSST protocol, participants sat quietly and saliva samples were collected at 15 min (T1), 30 min (T2), 45 min (T3), and 60 min (T4) post-TSST. Participants also completed the Childhood Trauma Questionnaire (CTQ) after the last (T4) post-TSST saliva sample was collected. For covariate analysis, participants completed a demographic and health behavior questionnaire and a measure of social support. At completion of the protocol, the participants were debriefed about the goals of the study and compensated with a $100 gift card to a local food store.

### Childhood Trauma Questionnaire

The CTQ Version 3 was used to measure the nature and extent of exposure to childhood adversity (40). The CTQ contains 28 items that assess 5 subscales of adversity: emotional abuse, physical abuse, sexual abuse, emotional neglect and physical neglect. Respondents rate each item using a 5-point scale (1 = *Never True* to 5 = *Very Often True*). The CTQ has high test-retest reliability and good convergent validity, correlating with interview-based rating of childhood abuse and therapist ratings of abuse (40, 41).

### Social Support

The Brief Social Support Questionnaire (BSSQ) was used to measure the perception of both availability of and satisfaction with social support, using six common support scenarios. Cronbach alpha reliabilities range from 0.90 to 0.93 (42), while construct validity was demonstrated by comparing it to the Social Support Measure (42, 43). BSSQ has been shown to be associated with markers of CVD (44) and psychological health (45). Social support was considered as a covariate in statistical models.

### Health Behaviors

Health behaviors were measured by self-report. Participants completed questionnaires that asked them about the types, intensity, and frequency (past month and past 3 days) of physical activity they engaged in, the quality of their sleep, use of tobacco products and street drugs, and daily intake of caffeinated and alcoholic drinks. Health behaviors were considerate as covariates in statistical models.

### Heart Rate Variability

HRV was calculated from the 5-lead, 3 channel Holter ECG recordings that were continuously measured from 15 min prior to the TSST, and up to 60 min post-TSST. A Cardiac Science Burdick Vision 5L Holter System was used. R-R intervals affected by ectopic beats were excluded and interpolated by linear function using Kubios HRV analysis software (46). The spectral analysis was computed using fast Fourier transformation (FTT). The ECG recordings were time-stamped at baseline (a 15-min segment), at start and end of the TSST protocol (a 10-min segment), and at the four consecutive 15-min segments that corresponded with the saliva sampling. Each segment was further subdivided into 3-min intervals that were averaged per segment for subsequent analysis. Spectrum analysis was performed separately on each 180-s (3 min) subdivision of the R-R interval sequence using an FFT algorithm. The lowest frequency that can be nominally resolved from a 180-s record is f = 0.0055 Hz, which is approximately an order of magnitude lower than the lowest frequency of the HRV-LF band. Frequency domain analysis covered total power (0.0–0.5 Hz) (TP), low (0.04–0.15 Hz) (LF) and high (0.15–0.40 Hz) (HF) frequency components. The LF/HF ratio was computed as LF power/HF power and is usually interpreted as an indirect estimate of the balance between the sympathetic and parasympathetic branches of the ANS, with higher values implying either greater sympathetic or less vagal influence (28).

### Salivary IL-6

All participants collected a saliva sample using salivettes (Sarstedt, Inc., Newton, NC) at 5 time points during the TSST procedure. IL-6 was measured in duplicate using a high sensitivity ELISA kit (R & D Systems, Minneapolis MN). The minimum detectable level of IL-6 ranged from 0.016 to 0.110 pg/ml, with a mean of 0.039 pg/ml. The intra-assay precision was 6.9–7.4%, while the inter-assay precision was 6.5–9.6%.

### Statistical Analysis

Summary descriptive statistics for all outcome and predictor variables were examined for normality of the distribution. To correct for a skewed distribution a square root transformation was applied to the LF/HF ratio values and salivary IL-6 values were natural log-transformed. Hierarchical Linear Modeling (HLM) 7.0 software for computing multilevel models for change (47), based on full maximum likelihood estimation, was used to examine intra-individual and inter-individual differences in baseline and trajectories of change over the course of the procedure (i.e., T0–T4) in both LF/HF ratio and salivary IL-6. All preliminary analyses were performed using SPSS software package 25.0 (Chicago, IL).

Two-level HLMs were computed; one for each outcome variable (i.e., LF/HF ratio and IL-6). For the model investigating change in the heart rate variability (LF/HF ratio), Level 1 predictor variables included linear and quadratic time slope parameters. A quadratic term was included to capture the curvilinear nature of the LF/HF ratio response profile. At Level 2, predictor variables included those that varied between participants (e.g., demographic characteristics, childhood adversity). The HLM model for the IL-6 response included linear and quadratic slope as well as the time-varying measure of HRV (LF/HF ratio) as predictors at Level 1. At Level 2, childhood adversity factors were included as predictors of the IL-6 response to the TSST. The structure of the HLM models allowed for examination of the interaction between childhood adversity factors and the LF/HF-HRV linear change over time (i.e., CTQ × LF/HF-HRV × Time interaction), in addition to the main effects of childhood adversity and LF/HF-HRV on the IL-6 response to TSST. For both analyses, time was measured in minutes from baseline (before TSST), which was coded as “0.” A combination of the two levels resulted in a mixed model with fixed and random effects (47).

The HLM analysis for each of the outcome variables was performed in two stages. At the first stage, the potential effects of the demographic variables including participant's age, education, marital status, income, health behaviors (physical activity, caffeine intake, alcohol consumption, smoking, and medication), social support, co-morbidities, and BMI were examined. During the second stage, effects of childhood adversity factors (i.e., emotional, physical, sexual abuse, and emotional and physical neglect) were examined. These variables were standardized to aid in the interpretation. To achieve the most parsimonious models, variables that had an effect (at *p*-value < 0.10 to be more conservative) were included in the final models. In addition, the error terms for quadratic slopes and slope for LF/HF-HRV were constrained to 0 (i.e., were not allowed to randomly vary across individuals). The fit for the reduced models was comparable to the saturated models based on the deviance test of the nested models and afforded greater statistical power (47). For the main HLM analysis alpha = 0.05, two-tailed test of significance was used.

## Results

### Descriptive Characteristics of Participants

Thirty four African American men (AAM), mean age 20 ± 2 years, were enrolled. Current demographic characteristics of the sample were previously reported (35). Briefly, 80% completed a high school education; 50% grew up in households with incomes <39 K/year; 56% had current incomes <29 K and 35% were unemployed. No one in the sample reported the use of any prescription medication or co-morbid conditions. The average BMI for the sample was 25.4 ± 3.8 kg/m^2^.

### Mean Levels of CTQ, Salivary IL-6, and HRV Pre/Post TSST

The mean value (±SD) for the CTQ scales were as follows: physical abuse (M = 8.31 ± 2.98); emotional abuse (M = 7.57 ± 3.48); sexual abuse (M = 6.59 ± 3.81); physical neglect (7.83 ± 3.38); and emotional neglect (M = 10.10 ± 3.42). These values for the CTQ scores were similar to that reported for a large validation sample of healthy adult males (48). Table 1 provides a summary of the descriptive statistics for HRV indices, and salivary IL-6 across five assessment times from baseline to 60 min post TSST protocol.

**Table 1:** Descriptive data for salivary IL-6 and indices of heart rate variability (*N* = 34).

	**T0 - Baseline**		**During TSST**		**T1**		**T2**		**T3**		**T4**	
	**M**	**SD**	**M**	**SD**	**M**	**SD**	**M**	**SD**	**M**	**SD**	**M**	**SD**
IL-6 (pg/ml)	0.97	1.48	n/a	n/a	1.59	3.31	1.06	1.94	0.95	1.78	1.33	2.36
HF	3.36	0.39	3.14	0.42	3.25	0.45	3.29	0.41	3.35	0.43	3.34	0.42
LF	3.51	0.27	3.30	0.28	3.40	0.31	3.45	0.26	3.48	0.28	3.47	0.27
LF/HF Ratio	1.67	0.45	1.88	1.60	1.84	1.12	1.78	1.09	1.67	0.85	1.67	0.94
HR	64.35	8.67	74.58	1.39	68.76	10.89	65.19	9.56	65.10	8.57	63.75	8.64

*Values are mean (M) and standard deviation (SD). TSST, Trier Social Stress Test; IL-6, interleukin 6; HF, log high frequency spectral power (0.15–0.40 Hz); LF, log low frequency spectral power (0.04–0.15 Hz); HR, heart rate beats/per min; T0, baseline; T1, 15 min post TSST; T2, 30 min post TSST; T3, 45 min post TSST; T4, 60 min post TSST*.

### HLM Analysis of the HRV and IL-6 TSST Response

Participants' current demographic, social support, and health behavior variables were entered into the model separately, and to achieve a parsimonious model, only those variables that were associated (at *p* <0.10) with outcome variables (i.e., LF/HF, IL-6) in the first stage of the HLM analysis were included in the final models. No associations were observed between demographic variables (age, education, marital status, and income), or social support and the LF/HF or salivary IL-6 response to TSTT. Hence, these variables were omitted from the final models. From the set of health behaviors, self-reported level of physical activity was significantly associated with LF/HF response and was retained in the final model. Both BMI and level of physical activity were significant predictors of the IL-6 response and were included in the final model.

#### Heart Rate Variability Response

**Figure 1:**
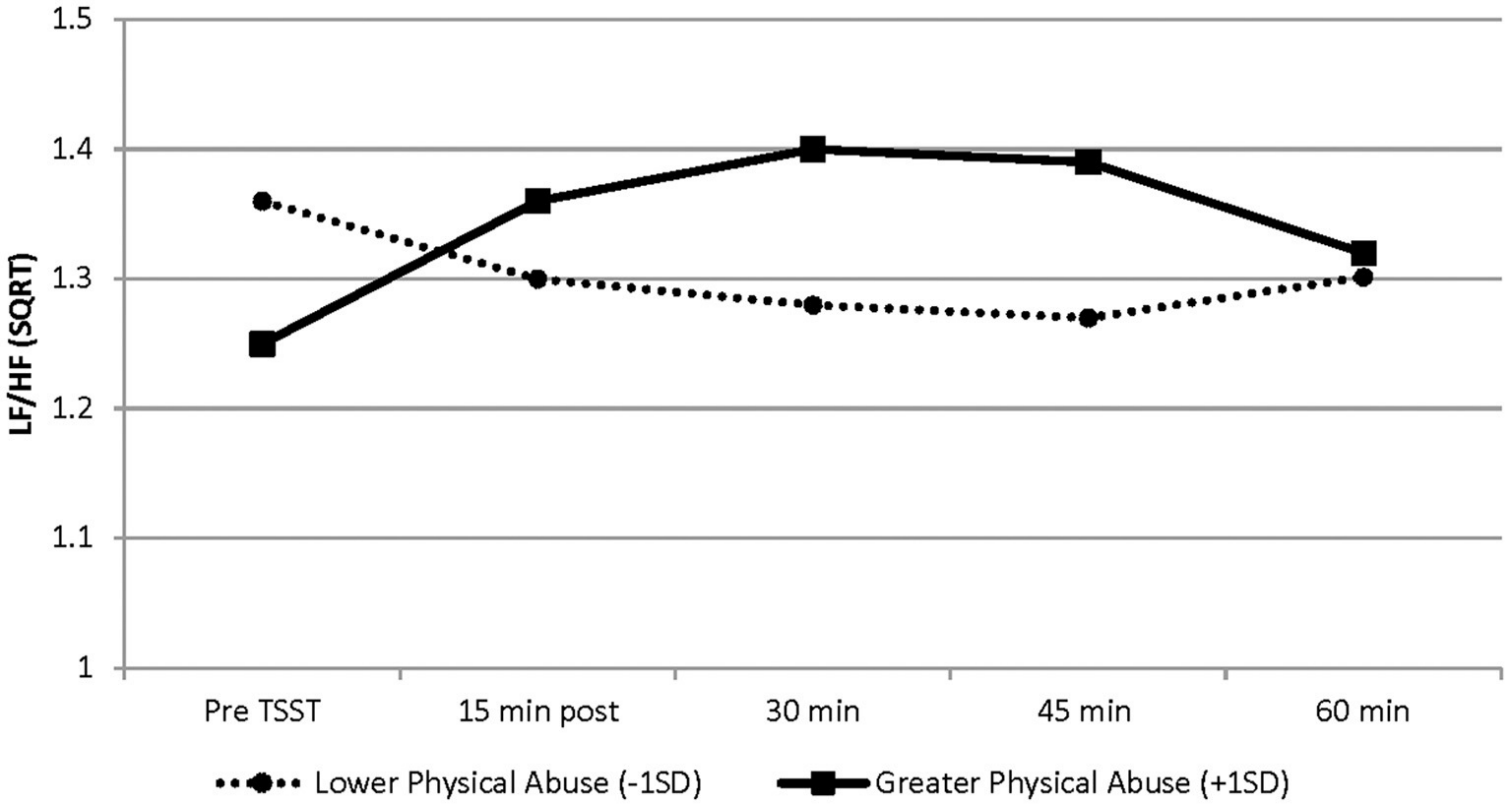
Graphical representation of the effects of physical abuse on LF/HF-HRV from baseline (before TSST) to 60 min post TSST. These effects are modeled as ±1 SD of the sample mean values on the physical abuse subscale. Greater exposure to physical abuse was associated with a steeper rise in the linear slope (b = 0.0086, SE = 0.0024, *p* = 0.001) and greater curvature of the quadratic slope (b = −0.00012, SE = 0.00003, *p* = 0.001). Solid line represents physical abuse subscale scores at +1 SD; dashed line represents physical abuse subscale scores at −1 SD. Graphs are estimated by the hierarchical linear models from baseline (before TSST) to 60 min post TSST. *N* = 34. SD, standard deviation; SE, standard error.

As shown in Table 2 and Figure 1, a significant association was found between the linear and quadratic rate of change in LF/HF ratio (from baseline to 60 min post TSST) and childhood physical abuse. Greater exposure to physical abuse was associated with a steeper rise in the linear slope (b = 0.0086, SE = 0.0024, *p* = 0.001) and greater curvature of the quadratic slope (b = −0.00012, SE = 0.00003, *p* = 0.001). Specifically, AAM with greater exposure to physical abuse exhibited a higher LF/HF ratio in response to the TSST, than men with lower exposure to physical abuse (see Figure 1). At baseline, physical abuse was not a significant predictor of LF/HF ratio (*p* = 0.073). No significant associations between HRV and physical or emotional neglect and emotional or sexual abuse were found.

**Table 2:** Final hierarchical linear model estimates for LF/HF ratio (*N* = 34).

	**Coefficient**	**SE**	***p*-values**
**Fixed effects (baseline** ^**a**^ **)**			
Intercept	1.3125	0.0862	0.000
Physical abuse	−0.1592	0.08538	0.073
Physical activity	−0.0799	0.0451	0.052*
**Time slope (linear)** ^**b**^			
Intercept	0.00085	0.00324	0.739
Physical abuse	0.0086	0.0024	0.001***
Physical activity	0.00048	0.0021	0.813
**Time slope (quadratic)**			
Intercept	−0.00003	0.00005	0.467
Physical abuse	−0.00012	0.00003	0.001***
Physical activity	0.000009	0.00004	0.795
**Random effect**			
Intercept		0.1653	<0.0001
Time slope		0.00001	>0.500
Level-1, e		0.0561	

*^a^Time was coded 0 at baseline to the original scale of measurement. ^b^Linear time slope intercept represents instantaneous rate of change at baseline. LF/HF values were square root transformed. At Level 1, Time variables were uncentered. The error term for the quadratic slope was constrained to zero, i.e., this coefficient was specified as fixed. At Level 2, predictors are either standardized or grand-mean centered.*

*p ≤ 0.05, ***p ≤ 0.001.

#### Salivary IL-6 Response

**Figure 2:**
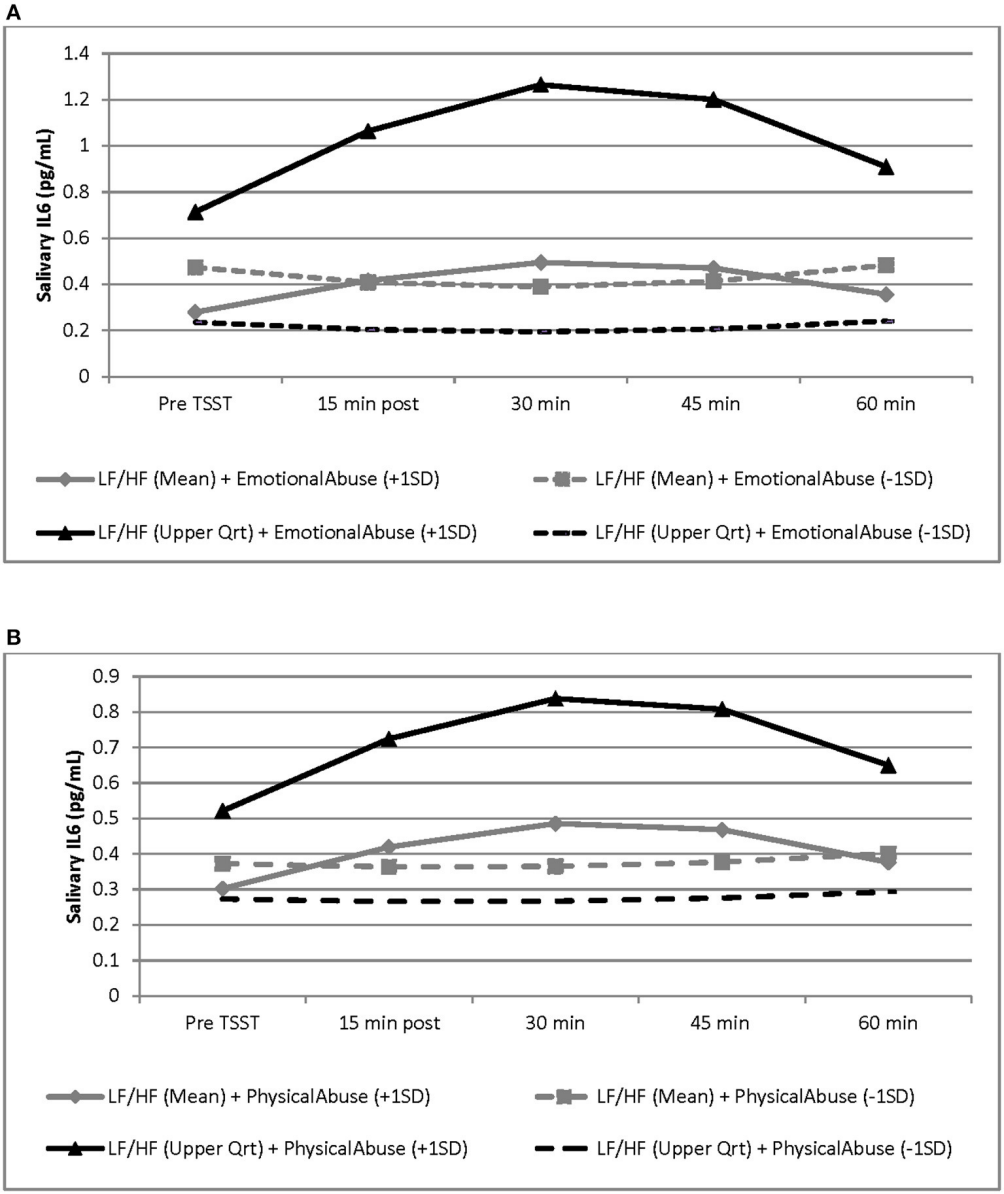
Graphical representation of the interaction between LF/HF-HRV and childhood adversity factors (i.e., emotional abuse and physical abuse) and the effects of these interactions on the salivary IL-6 response to the TSST. These effects are modeled as sample mean/upper quartile values for LF/HF ratio and as ±1 SD of the sample mean values for emotional and physical abuse subscales. Graphs are estimated by the hierarchical linear models from baseline (before TSST) to 60 min post TSST. *N* = 34. SD, standard deviation; SE, standard error. **(A)** For men with higher LF/HF-HRV, greater exposure to emotional abuse was associated with a more intense IL-6 response to the TSST, as compared to lower exposure to emotional abuse, and as compared to men with average LF/HF ratio (b = 1.7379, SE = 0.6396, *p* = 0.010). Black line represents LF/HF upper quartile values; gray lines represent LF/HF grand mean values; solid lines represent emotional abuse subscale scores at +1 SD; dashed line represents emotional abuse subscale scores at −1SD. **(B)** For men with higher LF/HF-HRV, greater exposure to physical abuse was associated with a more intense IL-6 response to the TSST, as compared to lower exposure to physical abuse, and as compared to men with average LF/HF ratio (b = 1.0746, SE = 0.3803, *p* = 0.007). Black line represents LF/HF upper quartile values; gray lines represent LF/HF grand mean values; solid lines represent physical abuse subscale scores at +1 SD; dashed line represents physical abuse subscale scores at −1 SD.

Although there were no significant main effects of CTQ subscales and LF/HF-HRV on the IL-6 response, results revealed a significant interaction between emotional abuse and LF/HF-HRV variation (b = 1.7379, SE = 0.6396, *p* = 0.010), as well as a significant interaction between physical abuse and LF/HF-HRV variation (b = 1.0746, SE = 0.3803, *p* = 0.007) during the TSST protocol (see Table 3). Specifically, the conditional relationship between childhood abuse and LF/HF-HRV indicated, that greater exposure to emotional and physical abuse combined with higher LF/HF-HRV across the TSST protocol was associated with a more intense IL-6 response as compared to men with lower exposure to emotional and physical abuse. In addition, for those with lower exposure to childhood abuse, the variation in LF/HF-HRV during the protocol was not associated with IL-6 response to the TSST. At baseline, there were no significant associations between emotional and physical abuse and IL-6 levels. No associations were found between physical/emotional neglect or sexual abuse and IL-6 response. See Table 3 and Figures 2A,B for graphical representations of these results.

**Table 3:** Final hierarchical linear model estimates for salivary IL-6 (*N* = 34).

	**Coefficient**	**Standard error**	***p*-value**
**Fixed effects (baseline** ^**a**^ **)**			
Intercept	−1.1743	0.3089	0.001
Emotional abuse	−0.0145	0.5637	0.980
Physical abuse	−0.1573	0.5461	0.779
BMI	0.0644	0.0697	0.366
Physical activity	−0.2144	0.2554	0.411
**Time slope (linear)** ^**b**^			
Intercept	0.0154	0.0107	0.166
Emotional abuse	0.0098	0.0255	0.704
Physical abuse	−0.0013	0.0198	0.948
BMI	−0.0009	0.0022	0.670
Physical activity	0.0182	0.0085	0.046*
**Time slope (quadratic)**			
Intercept	−0.0002	0.0002	0.196
Emotional abuse	−0.0002	0.0003	0.614
Physical abuse	0.00005	0.0003	0.844
BMI	0.000001	0.00003	0.976
Physical activity	0.00024	0.00012	0.051
**LF/HF ratio slope**			
Intercept	0.0111	0.2903	0.970
Emotional abuse	1.7379	0.6396	0.010**
Physical abuse	1.0746	0.3803	0.007**
BMI	0.1887	0.0632	0.005**
Physical activity	−0.4539	0.2079	0.035*
**Random effect**			
Intercept		1.6456	0.0001***
Time slope		0.0003	0.0001***
Level-1, e		0.2227	

*^a^Time was coded 0 at baseline to the original scale of measurement. ^b^Linear time slope intercept represents instantaneous rate of change at baseline. IL-6 values were natural-log transformed. At Level 1, Time variables were uncentered, SQRT LF/HF was grand-mean centered. At Level 2, predictors are either standardized or grand-mean centered. The error term for quadratic and LF/HF slopes was constrained to zero.*

*p ≤ 0.05, **p ≤ 0.01, ***p ≤ 0.001.

## Discussion

In this study we explored the relationships between exposure to childhood adversity, ANS activity and the proinflammatory (i.e., IL-6) response to an acute stressor. We hypothesized that childhood adversity would be associated with an altered ANS response to stress; and, further, that LF/HF-HRV would influence the IL-6 response to acute stress. Our findings revealed a direct relationship between physical abuse and the change in LF/HF-HRV, such that individuals with higher exposure to physical abuse showed a higher steeper rise in LF/HF-HRV response to the TSST. LF/HF ratio is a HRV index that provides an indirect assessment of ANS activity, with higher LF/HF ratio suggesting greater levels of sympathetic modulation and/or less vagal influence in response to the TSST. This observation is in accord with previous research wherein those with a background of neglect or abuse had diminished high-frequency (HF)- HRV and reduced parasympathetic activity (18, 49). Evidence also shows that the effects of childhood maltreatment on the ANS are long lasting (i.e., stable alterations) (50, 51). Findings from a large-scale study of adults (ages 33–79 years) suggests that the association between cumulative exposure to adverse life events and HF-HRV is not robust (52). However, in that study the investigators measured exposure to threatening experiences over the lifespan, as opposed to measuring exposure to adverse experiences during sensitive developmental periods (i.e., early life) when stress response systems are more malleable (53). Thus, our findings add to the collective evidence, that childhood adversity embeds enduring alterations in the ANS stress response pathways (50, 51, 53).

Our findings further demonstrate that for participants with greater exposure to physical abuse or emotional abuse, a higher LF/HF-HRV predicted a greater IL-6 elevation in response to the TSST stress task; while for men with lower exposure to abuse, variation in LF/HF-HRV was not associated with IL-6 response. A lack of significant main effects of exposure to childhood adversity and LF/HF-HRV on the IL-6 response indicates that there is a conditional relationship between these factors. Specifically, the interaction suggests a predisposition for an increased proinflammatory stress response for men who had greater exposure to abuse coupled with a higher LF/HF-HRV (i.e., greater sympathetic activity relative to parasympathetic activity).

Causal evidence in animal models demonstrates that inflammatory processes are adaptively regulated by the vagus nerve through the cholinergic anti-inflammatory pathway, such that stimulation of the vagus inhibits proinflammatory cytokine release (20). This “Inflammatory Reflex” is activated by inflammatory mediators released from peripheral tissues that trigger firing of vagal nerve fibers, which dampen inflammation (22–24, 27, 32, 54). Evidence for the cholinergic anti-inflammatory pathway in humans has been described. For example, Marsland et al. demonstrated that high-frequency HRV, reflective of parasympathetic activity, was associated with lower *ex vivo* stimulated production of the proinflammatory cytokines, TNF-alpha and IL-6 (30). Also, using RR interval variability as an index of cardiac vagal modulation, Sloan et al. reported a strong inverse relationships between cardiac vagal activity and levels of CRP and IL-6 in young adults; supporting the hypothesis that reduced descending vagal anti-inflammatory signals can lead to cytokine overproduction in humans (32). Consistent with this, the association of adverse childhood experiences and inflammation has been shown to be mediated by greater sympathetic nervous system activity, as indexed by higher urinary norepinephrine output (55). A meta-analysis of human studies evaluating HRV and inflammation identified several studies reporting a negative relationship between HRV and inflammation, supporting the concept that indices of HRV (i.e., HF and SDNN) reflect activity of the neurophysiological pathway involved in adaptive regulation of inflammatory processes in humans (34). The findings of the present study, therefore, add to the evidence that for individuals with childhood adversity, an altered ANS system response to stress contributes to a greater stress-induced inflammation in adults.

For this investigation, salivary IL-6 was measured as an indicator of stress-induced inflammation. We previously observed elevations in salivary IL-6 in response to the TSST in postmenopausal women who reported higher levels of perceived discrimination (56). Likewise, others report elevations in salivary IL-1 beta in response to an acute laboratory stress paradigm (57). Elevations in salivary inflammatory markers have clinical relevance, as elevated salivary CRP levels have been associated with increased CVD risk (36). Local inflammation may also provoke emotional states, similar to sickness behavior, and characterized by depressed mood, fatigue, and social withdrawal (58). Similar to inflammatory-vagal afferent brain signaling (58), inflammatory molecules within the oral environment may initiate afferent trigeminal nerve activation to alert the brain and alter emotional and behavioral states (58–61). Persistent elevations of proinflammatory cytokines in response to stressful challenge may foster and/or perpetuate a negative emotional state, and risk for mental health disorders.

Previous studies, which assessed the relationship between childhood adversity and the response to stress, typically group all forms of childhood adversity as a whole (8, 62–66). In contrast, we analyzed the five subtypes of adversity assessed by the CTQ (emotional abuse, physical abuse, sexual abuse, emotional neglect, and physical neglect) separately. Significant associations were found only with physical abuse and emotional abuse. These findings may shed light on differences in the relationships of exposure to childhood abuse vs. childhood neglect to ANS regulation and proinflammatory response to acute stress. Differences in the effect of abuse and neglect may also be attributed to dissimilarities in the long-term effects of these two forms of maltreatment.

The current study holds a number of limitations. First, we examined a small number of men, restricting the number of predictors that we could investigate without compromising statistical power. Also, our sample consisted of healthy young men; and, thus, cannot be generalized to women, older adults, or those with existing CVD. Yet, this age group is an important group to study, as earlier identification of stress-related inflammatory risk can allow early targeting of interventions to this vulnerable group, prior to manifestation of inflammatory-based disease (i.e., CVD), which have worse outcomes for African American men (1). Because of the correlative nature of this study, the findings should not be interpreted to indicate that exposure to childhood adversity has a direct causal effect on the physiological and inflammatory profiles of AAM. Instead the present results support the existence of an association between these factors. Further, given the small sample size, current findings warrant further investigation with a greater sample size. Finally, although HRV is regarded as an index of ANS activity, the physiological interpretation of the spectral components (LF, HF) and the statistical derivatives (e.g., LF/HF ratio) calculated from the beat-to-beat fluctuations are debated. While the majority, but not all (67–69), agree that the efferent vagal activity is a major contributor to the HF power, the interpretation of the LF component is less definitive. In addition, the complex non-linear reciprocal changes in parasympathetic and sympathetic activity influence the LF/HF ratio limits interpretation of the LF/HF ratio as an indirect measure of sympatho-vagal balance (70, 71).

## Conclusions

This investigation suggests childhood abuse to associate with altered ANS response to stress, and that greater exposure to childhood abuse in combination with altered ANS reactivity may contribute to a greater proinflammatory response to acute stress. ANS reactivity is thought to reflect one's global capacity to produce an adaptive psychophysiological response to stressors (72). Although hypothetical, a lower LF/HF-HRV in individuals exposed to greater childhood adversity may buffer the inflammatory response to acute stress challenge, promoting better adaptation and perhaps stress resilience in the face of adversity (73). These findings are preliminary but support the need for studies using a larger sample size and more direct measures of ANS activity.

## Data Availability Statement

The raw data supporting the conclusions of this article will be made available by the authors, without undue reservation.

## Ethics Statement

The studies involving human participants were reviewed and approved by the Loyola University Chicago Institutional Review Board for the Protection of Human Subjects and informed consent was obtained from all participants. The patients/participants provided their written informed consent to participate in this study.

## Author Contributions

LJ, HM, and RB contributed to conception and design of the study. DT collected data, organized the database, and performed the statistical analysis. DT, RB, and HM wrote sections of the manuscript. All authors contributed to manuscript revision, read, and approved the submitted version.

## Funding

This investigation was supported in part by the President's Intercampus Collaborative Research Stimulation Awards – Loyola University Chicago. DT was supported by NIH R01 CA125455-02S1 supplement awarded to LJ and HM. Also, partial support for this project was obtained from the Ruth K. Palmer Memorial Endowment: a gift of Dean Emeritus Gladys Kiniery in memory of her beloved sister.

## Conflict of Interest

The authors declare that the research was conducted in the absence of any commercial or financial relationships that could be construed as a potential conflict of interest.

## Publisher's Note

All claims expressed in this article are solely those of the authors and do not necessarily represent those of their affiliated organizations, or those of the publisher, the editors and the reviewers. Any product that may be evaluated in this article, or claim that may be made by its manufacturer, is not guaranteed or endorsed by the publisher.
